# The strength of domestic production networks: an economic application of the Finn cycling index

**DOI:** 10.1007/s41109-021-00411-5

**Published:** 2021-09-19

**Authors:** Erik Braun, Tamás Sebestyén, Tibor Kiss

**Affiliations:** 1 Faculty of Business and Economics, University of Pécs, Pecs, Hungary; 2 Institute of Regional Studies, Eötvös Loránd Research Network, Centre for Economic and Regional Studies, Pecs, Hungary; 3 MTA-PTE Innovation and Economic Growth Research Group, Pecs, Hungary; 4 Blue Economy Research Centre, Pecs, Hungary

**Keywords:** Production network, Cycling, Input–output network, Economic structure, Global value chains

## Abstract

There has been an increasing interest in analyzing the structure of domestic and global supply chains/networks in the past decade. Concerns about potential (systemic) risks resulting from overdependence on global supply networks have been magnified during the lockdowns triggered by the COVID-19 pandemic in the last year. Strengthening local and/or domestic networks may be an adequate approach to overcome the severe economic implications of this overdependence, but it also rises the question of how one can measure the strength of domestic supply/production networks and design an appropriate structure. The objective of this paper is to propose a method for measurement and to provide a first-cut analysis with this method on a sample of economies. Building on ecological network analysis, we borrow the Finn cycling index from its toolbox and show a ranking of countries with respect to the strength of their domestic production networks based on this index. The results suggest that the countries are very heterogeneous both in terms of the level of cycling index and its sectoral decomposition. Using panel-econometric techniques, we point out the role of the openness and structural asymmetry in shaping this strength, also controlling for other macroeconomic characteristics of the economies. The estimates reveal that openness has a negative, while asymmetry has a positive effect on this index, but other country-specific characteristics also play a role in shaping the systemic operation of national economies as measured by the Finn cycling index.

## Introduction

There has been a wide discussion about the overly fragile nature of global supply chains/networks. While this highly optimized global system brings efficiency to production activities, it also builds up systemic risk which can result in harsh disruptions in and fallback of economic activity. The economic consequences of the COVID-19 pandemic provide a recent and powerful example of this fragility, putting this discussion into the focus of attention. In order to avoid overwhelming their healthcare systems, many countries put constraints on economic activity by implementing sectoral and transportation lockdowns. Restaurants and theatres have been closed, mass events banned, traveling between countries restricted, and companies closed where workers were found infected. As a result of these arrangements, tourism collapsed rapidly, international shipping stalled, and access to the inputs became more difficult in the global market.

Due to the wide interconnectedness of countries, global value chains conveyed the economic effects of domestic lockdowns over borders (Barrot et al. 2020; Fang et al. 2020; Guan et al. 2020). The World Trade Organisation (WTO 2021) reported a 14% decline in the volume of bilateral trade in the first two quarters of 2020 (8,000,671 million US dollars) compared to the previous year (9,307,784 million US dollars), confirming disturbances in global value chains. Indeed, Vidya and Prabheesh (2020) find a significant drop in trade interconnectedness and a structural change in global value chains due to the pandemic, which may also lead to a decrease in demand (Baldwin and Freeman 2020). On the other hand, Barrot et al. (2020) and Guan et al. (2020) show that the intensity to which these shocks affect a given economy depends on the structure of the domestic production networks and the exposure to foreign markets (Barrot et al. 2020; Guan et al. 2020).

French minister of finance, Bruno le Maire also shed light on the problems arising from disruptions in global value chains and economic lockdowns emphasizing that *“supply problems create strategic problems in certain industries”* and pointing to the over-dependence of the French economy on Chinese imports: according to his report, 80% of the inputs of the French pharmaceutical industry comes from China (Reuters 2020). Due to such over-dependence, domestic economies face serious risks from disruptions in the global value chains.

These economic effects of the COVID-19 pandemic provide an example of how the globally optimized production networks are vulnerable to shocks of this kind and it brought into the focus the concerns about the ultimate efficiency of globally organized value chains and production networks, while putting in context the recently strengthening discussion about renationalizing, i.e. revitalizing local or domestic supply chains (Bonadio et al. 2020). As the French minister of finance himself argued, strengthening these local networks can be an adequate approach to overcome the severe economic implications of future lockdowns. However, such initiatives as backshoring, nearshoring (Piatanesi and Arauzo-Carod 2019) or renationalization (Bonadio et al. 2020) can not unconditionally lead to a less vulnerable economy because of the higher risk arising from domestic lockdowns in this case (Bonadio et al. 2020).

At least two important questions arise from this agenda. First, where is the optimal trade-off between efficiency gains from globally optimized but fragile production networks and lower risk resulting from more localized but redundant supply chains? Second, what methodological tools can be fruitfully applied in answering the former question, in particular how one can measure the strength of local supply networks? This paper contributes to the second question by proposing a way to measure the strength of domestic supply or production networks.

In this attempt, our contribution is linked to previous literature in several interrelated areas. First, a series of recent studies have analyzed the macroeconomic impact of the pandemic by introducing an epidemiological approach into economic models (Acemoglu et al. 2020; Atkeson 2020; Alvarez et al. 2020; Eichenbaum et al. 2020; Glover et al. 2020; Krueger et al. 2020) while more standard input–output models have also been applied to reveal the extent to which economic activity can decline due to economic lockdowns. These papers show that the extent of the economic downturn depends on the sectoral composition of the economies (Bonadio et al. 2020), on the duration of lockdowns and the number of countries that implement them (Guan et al. 2020). Indirect linkages seem to play a crucial role in spreading the shocks Giammetti et al. (2020).

Even before the pandemic, several studies have examined the structure and the shock propagation capability of global supply chains in many ways. With the help of network analytic tools, the central actors can be identified in supply chain networks, and it is possible to track down how shocks propagate between them (Cerina et al. 2015; Fan et al. 2014). The available multi-sector and multi-country datasets have allowed the examination of the structure of trade in value-added (Amador and Cabral 2017), detecting the community structure (Cerina et al. 2015), and revealing the countries’ positions in production stages by sectors (Cingolani et al. 2017). In addition, a series of recent studies have investigated global supply chains as a multilayer network where countries/regions are the nodes and the sectors are the network layers while links represent trade flows (Alves et al. 2019; Gomez et al. 2020; Coquidé et al. 2020). These studies point to the potential in network analysis as a useful tool in measuring different aspects of the structure of global production networks or supply chains. This approach can be extremely useful for analyzing the possible benefits from more locally organized production networks which can be a structural manifestation of recent developments already pointing towards a slowdown in international trade and strengthening protectionism (Coe and Yeung 2019).

In the economic literature, economic structure as described by intersectoral transactions is analyzed with a range of different input–output models (Miller and Blair 2009). These studies include, among other things, the measurement of value-added in trade (Timmer et al. 2014; Johnson and Noguera 2012; Koopman et al. 2014) the length of domestic and global production/supply chains (Wang et al. 2017), identifying the central sectors of an economy (Giammetti et al. 2020), and examination of the sectoral effects of disasters (Dietzenbacher and Miller 2015). The application of network theory methods complements these standard models and more complex analysis can be performed by mixing these techniques (Amador and Cabral 2017).

With respect to the intersectoral structure of the domestic economy, previous studies have shown that the asymmetric structure of transactions increases the volatility of aggregate output (Acemoglu et al. 2012). Input–output linkages play a crucial role in transmitting shock between economic actors (Carvalho et al. 2020) and that central sectors in the transaction network are primarily responsible for the amplification of idiosynchratic shocks (Contreras and Fagiolo 2014). While these results are important in understanding the shock propagation mechanism within an economy, no study, to our knowledge, has examined so far the level or extent of the systemic operation of an economy or in other terms, the overall strength of its internal production network. Building on ecological network analysis, we borrow the Finn cycling index from its toolbox. This index measures the ratio of cycling or in other terms the extent to which feedback loops are present in a system. Representing system elements with nodes in a network, cycling reflects the direct and indirect connections (feedback loops) that link together these nodes. System elements are economic sectors in our exercise, thus cycling refers to the extent to which these sectors are connected by relatively strong input–output linkages creating loops in the system, allowing for a less hierarchical organization of production processes. Ecological network analysis has been used earlier for similar purpose: Kharrazi et al. (2013) analysed six economic resource trade flow networks with the help of this methodology and Alves et al. (2019) examined the nestedness of countries in international trade flow networks by sectors. While the cycling index is a good candidate for our purposes, we also show that the asymmetry of production network structure, economic openness and other macroeconomic characteristics of the economies shape the value of this index. Using panel-econometric techniques, we point out that by controlling for these characteristics, one can estimate the pure contribution of the network structure behind domestic economies to the cycling index.

The objective of this paper is to propose the Finn cycling index as a method of measuring the level of systemic operation in economies and to provide a first-cut analysis on a sample of economies using the proposed tool. In addition to ranking a set of countries with respect to the strength of their domestic production networks, we also analyze the role of asymmetric network structure and the openness of the economy in shaping this strength.

The paper is structured as follows. In “Data and methods” section, we give a brief discussion of the data we use and the methodological background of the analysis with special attention to measuring the cycling index. In “Results” section, first, we show the main characteristics, such as openness and asymmetry of production networks, and then, we rank the countries by cycling index. At the end of the section, we estimate the role of structural and macroeconomic properties in shaping the cycling index. Finally, in “Discussion and conclusion” section, we conclude the results, describe the limitations, and pins down some avenues for future research.

## Data and methods

In this section we first describe the logical framework of the study. We present the schematic model of the production networks that we use, introduce the Finn cycling index as a candidate of measuring the strength of domestic networks or production feedback loops and show how it can be interpreted in some special cases. Then, we describe the data we use in the remainder of the paper to feed the model.

### The production network

The production of final goods takes place in several steps, typically along vertically differentiated supply or value chains, where these production stages may be geographically dislocated as a result of exploiting comparative advantages (Cingolani et al. 2017). On the other hand, these value chains behind different final goods has been merged into a complex global production network, the structure which heavily affects how countries and sectors participate in the international division of labor and specialization. A series of recent studies emphasized the rising importance of global value chains in analyzing international trade (Baldwin and Lopez-Gonzalez 2015; Johnson and Noguera 2012; Timmer et al. 2014). While early papers focused on mapping the structure and dynamics of these networks, more recent contributions try to explain network formation (Coe and Yeung 2019).

In contrast to most of these efforts which take a global perspective, we use a different approach in this paper and investigate the structure of domestic production networks. The idea behind this focus is that strong specialization in global production networks reduces the chance that domestic economies can properly function in case the global transactions stall in an abrupt manner. This means that instead of analyzing the whole global transaction network or input–output table, we slice it into separate domestic input–output tables. The schematic setup of these tables are represented in Table 1. The unit of the analysis is an economic sector, of which we can have an arbitrary but finite number *N*. The $x_{i}$ total output of sectors *i* in a given country is purchased by other sectors *j* (denoted by $w_{ij}$ in general), by final users within the country (households, government and firms, denoted by $z_{i}$) or exported to other countries (labelled by $y_{i}$). To produce output $x_{i}$, Sector *i* uses inputs from other domestic sectors *j* (denoted by $w_{ji}$) and from imports (denoted by $m_{j}$). In addition to these expenses, sector *i* pays income to production factors, indirect taxes and transport margins which are included in the row of value added for simplicity (labelled by $v_{j}$). As the latter is assumed to include all expenses in addition to intermediate inputs, the row- and column-sums must equal: $\sum _{j} w_{ij} + z_{i} + y_{i} = \sum _{j} w_{ji} + v_{i} + m_{i} = x_{i}$.

**Table 1:** A schematic domestic production (input–output) network

	Sector 1	Sector 2	…	Sector N	Final use	Export	Total output
Sector 1	$w_{11}$	$w_{12}$	…	$w_{1N}$	$z_{1}$	$y_{1}$	$x_{1}$
Sector 2	$w_{21}$	$w_{22}$	…	$w_{2N}$	$z_{2}$	$y_{2}$	$x_{2}$
…	..	…	…	…	…	…	…
Sector N	$w_{N1}$	$w_{N2}$	…	$w_{NN}$	$z_{N}$	$y_{N}$	$x_{N}$
Value added	$v_{1}$	$v_{2}$	…	$v_{N}$			
Import	$m_{1}$	$m_{2}$	…	$m_{N}$			
Total output	$x_{1}$	$x_{2}$	…	$x_{N}$			

The most important element of the analysis in this paper is the matrix ${\mathbf {W}}$ which collects transaction volumes $w_{ij}$, thus represent the weighted adjacency matrix between domestic sectors. Hence, nodes in the production network are economic sectors while edges are transactions measuring the value of goods and services supplied from sector *i* (in the rows) to sector *j* (in the columns). The second important ingredient is the vector ${\mathbf {m}}$ containing the import volumes $m_{i}$ of every sector, thus reflecting the input-side foreign exposure of the sectors.

### Finn cycling index

The Finn cycling index (Finn 1976) is capable of describing the strength of internal feedback loops within the domestic economy. It measures those processes, which go back to their original places (sectors) either directly or indirectly (Fath 2015). The cycling index “denotes how many times further than the straight throughflow path length an average unit of inflow travels because of cycling …” (Finn 1976, p. 369). There are more variations of this index (Allesina and Ulanowicz 2004; Kazanci et al. 2009), but the original index is still generally used.

The starting point for this index is matrix ${\mathbf {W}}$, containing raw transaction volumes between the domestic sectors. These transaction volumes are normalized column-wise with the sum of the given column to obtain input–output coefficients which represent the share of sector *i* in contributing to sector *j*’s total input. This share accounts for other domestic sources as well as imports and value added (in the broad sense as used above):

1 $$\begin{aligned} a_{ij} = \frac{w_{ij}}{x_{j}} \end{aligned}$$

This information is structured in matrix ${\mathbf {A}}$. The normalization have two consequences. First, we lose information on the mere size of the economy, i.e. results from now on become insensitive to the absolute values of sectoral outputs. Second, we keep information on the external exposure (openness) of the economy: the more important is the import for a given sector, elements in ${\mathbf {m}}$ will be higher compared to ${\mathbf {x}}$, thus matrix ${\mathbf {A}}$ will have lower values on average.

From the input–output coefficients in ${\mathbf {A}}$, we calculate the following Leontief-inverse:

2 $$\begin{aligned} {\mathbf {L}} = ({\mathbf {I}}-{\mathbf {A}})^{-1} \end{aligned}$$

where ${\mathbf {I}}$ is the identity matrix. The general element $l_{ij}$ of the Leontief-inverse matrix shows the overall connectedness between sectors *i* and *j* either directly, as in ${\mathbf {A}}$, or indirectly through other sectors. This can be formally shown by the matrix-power expansion of the given inverse. As a result, $l_{ij}$ measures the relative strength of feedback loops of different lengths between sectors *i* and *j*.

Next, we pick the diagonal elements of the Leontief-inverse in Eq. 2 and normalize them according to:

3 $$\begin{aligned} {\hat{l}}_{i} = \frac{l_{ii}-1}{l_{ii}} \end{aligned}$$

Using the previous definition of the elements of the Leontief-inverse, the diagonal elements reflect the strength of feedback loops around a given sector (summing over loops of different lengths). As the structure of the Leontief inverse ensures that we have diagonal elements larger than one, it follows that the normalized values in Eq. 3 reflects the relative strength of the feedback loops around a given sector—thus we may call them sector-level cycling indices (Finn 1976). In other terms, these values show the extent to which sector *i* contributes to the overall production of the economy, reflecting how strongly the given sector is embedded in its systemic operation. Finally, we calculate the weighted average of these sector-level cycling indices in order to obtain an economy-level indicator:

4 $$\begin{aligned} FCI = \frac{1}{\sum _{i} x_{i}} \sum _{i} {\hat{l}}_{i} x_{i} \end{aligned}$$

Some notes about the economic application of the Finn-index. Originally, in ecology, the on-diagonals are all zeros, because the own consumption of compartments are handled as waste (e.g. respiration, energy loss) and as such, they place it to the right hand side [see e.g. the original Finn’s tables (Finn 1976), Allesina and Ulanowicz (2004) or Szyrmer and Ulanowicz (1987)] . However, in economies these tables contain the intrasectoral transactions (self-loops). As a consequence, while in ecology $l_ii$ values contain a “clean” intersectional relationship, in economy $l_ii$ values also contain the intrasectoral transactions. One solution is to use the net input–output tables, where diagonal elements are set to zeros. The construction of the net-tables can be performed by the method, proposed by (Miller and Blair 2009): remove the diagonal elements and reduce the outputs ($z_i$) accordingly. However, intrasectoral transactions describe links between firms belonging to the same sectors. In other words, these transactions are inter transaction at a lower level of economic aggregation. Therefore, similar to other studies (see e.g. Alves et al. 2019; Giammetti et al. 2020), it is reasonable to work with the gross input–output tables. In this study, we will use the gross tables everywhere.

For further reference, we introduce the variable import-openness, which characterizes every sector specifically and denotes the share of imports within its overall input (not counting value added):

5 $$\begin{aligned} \tilde{m}_{i} = \frac{m_{i}}{\sum _{i}w_{ji} + m_{i}}, v_{i} = 0 \end{aligned}$$

We used Eq. 5 to calculate sector-level input (import-) openness for every country, sector and year. Then, country-level openness is obtained by the weighted average of sector-level openness indicators, where the weights are sectoral outputs ($x_{i}$). For asymmetry, we took the relative input shares in matrices ${\mathbf {A}}$ for all countries and years, and calculated the skewness (third moment) on the observed distribution of these input shares. By out-degree centrality, we mean the sum of rows in ${\mathbf {A}}$ ($\sum _{j}a_{ij}$), and it shows the importance of a given sector as supplier of other domestic sectors. As we have a weighted network with non-zero values at almost all entries, we measure the density by the average (weighted) out-degree centrality (average strength) of the nodes.

### Some special cases

In this subsection we illustrate the cycling index through a few special cases. First, we provide analytical results for two very extreme cases (empty and completely connected economies), then three numerical examples show how loops shape the value of the index. Finally, we demonstrate the difference between the cycling indices calculated for symmetric and asymmetric (scalefree) random networks.

**Fig. 1:**
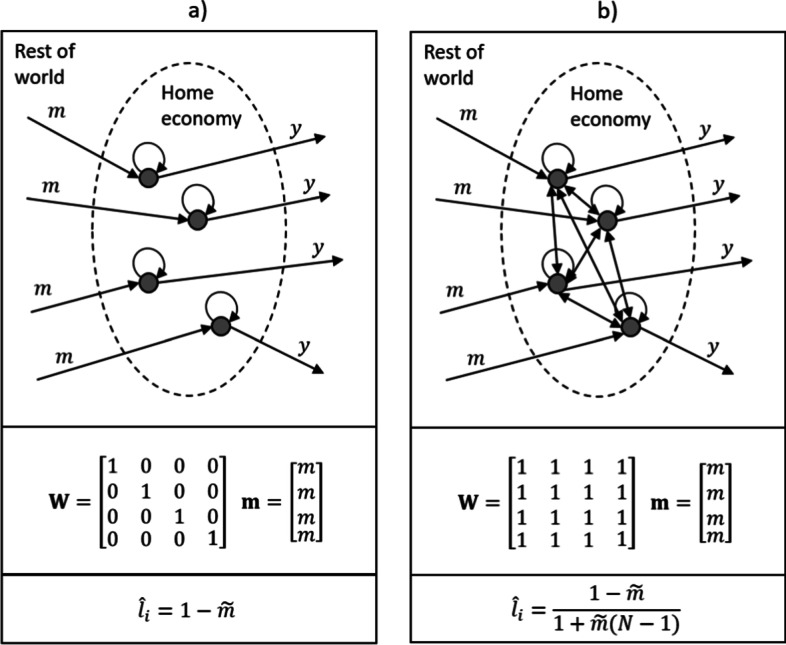
The schematic framework for a domestic production network, with two extreme cases. **a** is an economy with isolated activities but foreign exposure, **b** is a balanced economy with identical internal transaction volumes

The schematic structure of the model and the two extreme cases are represented in Fig. 1. Here, the dots represent domestic sectors, while the arrows show the transactions between them. The figure shows only four sectors, but the analytical result derived in the “Appendix 1” hold for an arbitrary number *N* of sectors. Panel (a) in Fig. 1 shows an atomistic economy, with no inter-sector transactions between the domestic sectors, but we allow for international exposure. For simplicity, intra-sector transactions are normalized to one. As shown in the “Appendix”, the cycling index in this case becomes ${\hat{l}}_i = 1-\tilde{m}$ for all sectors *i*. This means, that *given an isolated internal structure*, increasing openness have a negative effect on the cycling index, reflecting the idea that increasing foreign exposure decreases the independent operation of an economy.

Panel (b) in Fig. 1 reflects an economy with a completely balanced internal structure where intra-sectoral and inter-sectoral transactions have the same intensity. As shown in the “Appendix”, the sector-level cycling indices turn out to be $(1-\tilde{m})/[1+\tilde{m}(N-1)]$ in this case. An interesting point here is that the size of the economy in terms of the number of sectors becomes important, as reflected by *N* in this formula. Second, given that $N > 1$, openness still has a negative effect on the cycling index.

**Fig. 2:**
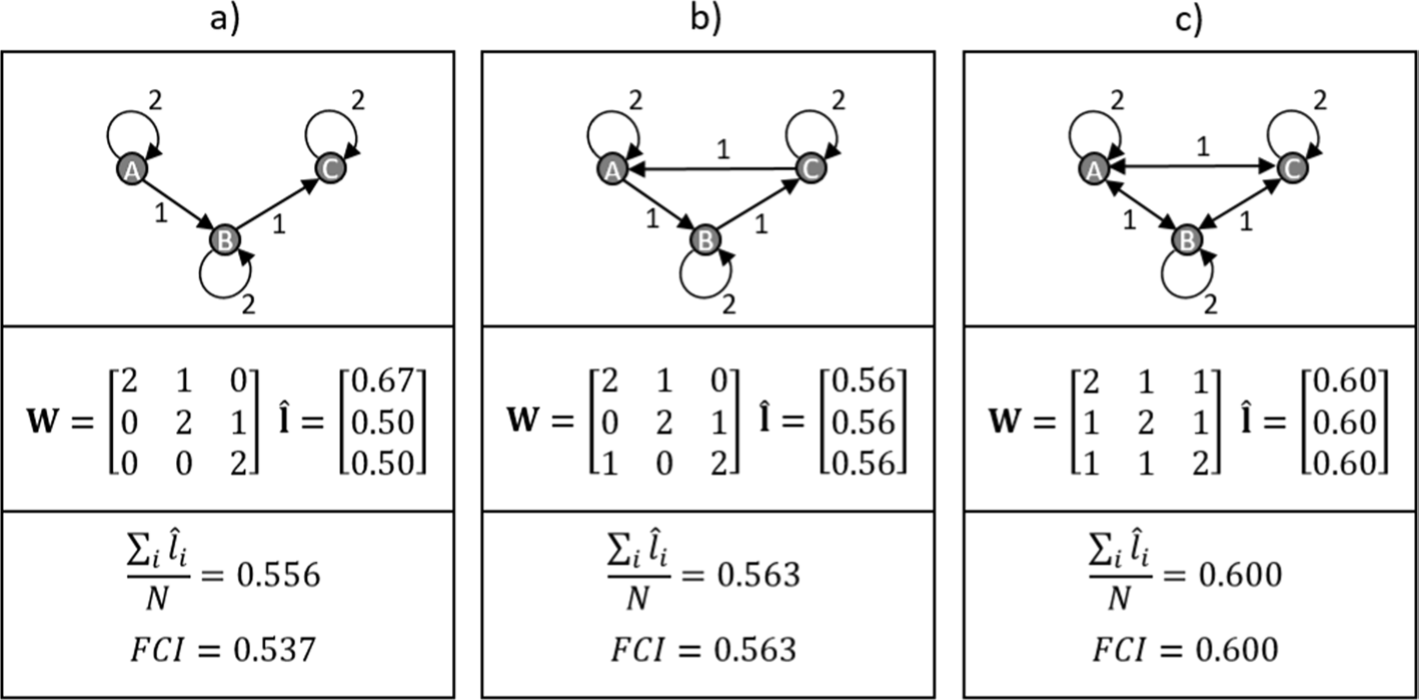
Three numerical illustrations with three sectors. **a** represents a no-feedback structure with a single production line. **b** represents a feedback loop in addition. **c** represents a completely balanced structure. The calculations assume unit value added or import volumes in all sectors

Beyond these two extreme cases, Fig. 2 represents a few illustrative calculations when there are only three sectors. In these examples we assume unit import plus value added volumes for all sectors, and concentrate on the effect of feedback-loops appearing in the system. In panel (a) we show a simple supply chain where sector A supplies sector B which then supplies sector C, but there is no feedback loop in the economy. The sector-level cycling indices are shown in vector $\hat{{\mathbf {l}}}$. These are asymmetric, reflecting the directed links in the system. Sector A has a higher score as it is the origin of the supply chain, thus it is less exposed. The simple average of these values is 0.556, while the weighted average (where weights are sectoral total outputs) is smaller as sector A has a lower output (remember that value added and import are assumed to sum up to 1, so total output of sector A is 3, while that of the other two sectors is 4).

Panel (b) represents a slight addition, by inserting a feedback loop between sector C and A, making the economy more circular. Sector A looses from its individual cycling score, while sectors B and C formerly gain at the end of the chain. The overall result is positive though, the *FCI* values become identically 0.563, which means an approximately 5% increase. This illustration shows that inserting feedback loops into the economic system typically increases the Finn cycling index as defined in Eq. 4, or in other terms contribute to the systemic operation of the economy.

Finally, panel (c) in Fig. 2 shows a situation where these feedbacks are maximized within the economy, i.e. it is established in both directions. Calculations show that the sector level cycling indices increase identically to 0.6, reflecting a 7% increase compared to the one-directional feedback loop in panel (b).

These illustrations nicely show that the proposed cycling index in the spirit of Finn (1976) reflects the capability of domestic economies to function systemically and shows the extent to which domestic sectors are able to contribute to each other’s productive activities. It turned out from the extreme cases that the level of foreign exposure clearly decreases the value of the index, while the number of sectors included is also important—a result which needs to be taken into account when comparing production networks of different size. In this study we do not face this problem as we compare production networks with a fixed size.

One may also investigate if the Finn cycling index depends on edge weights or the structure of the network. In order to have an insight in this respect, we constructed two sets of random networks. First, we simulated 100 independent random networks of the Erdős–Rényi type (Erdös and Rényi 1959) which shows a symmetric structure with relatively similar nodes. Then, we simulated 100 independent random networks with the Barabási–Albert algorithm (Albert and Barabási 2002) which shows an asymmetric, scalefree structure with power law degree distribution. Then, we calculated the Finn cycling index for all these networks according to Eq. 4 and averaged over the 100 independent networks for the two models separately.

**Fig. 3:**
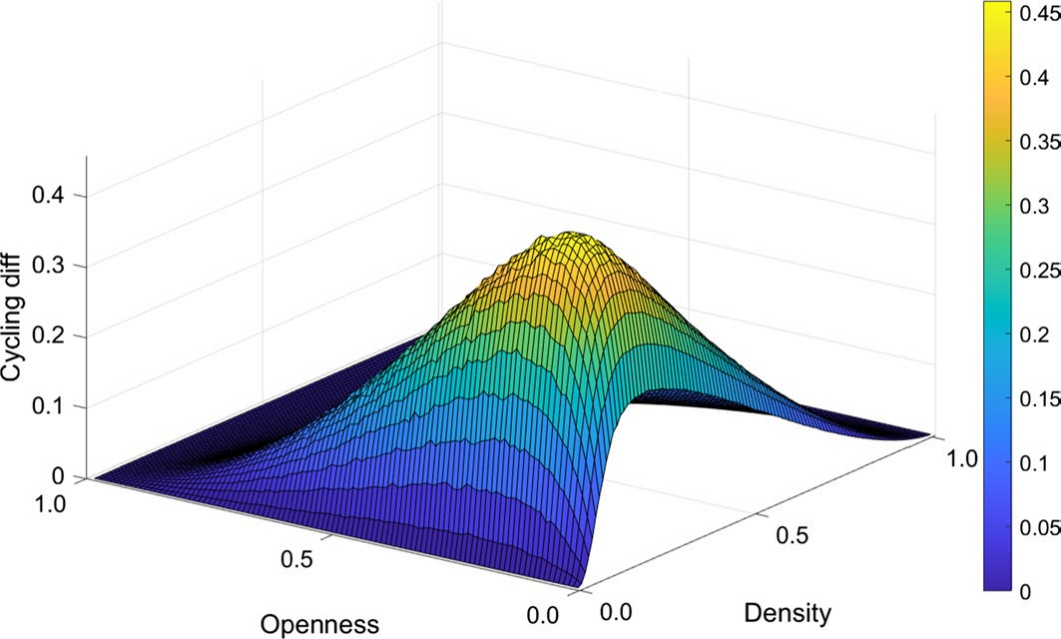
Cycling in symmetric and asymmetric (scale-free) random networks. The vertical axis shows the average difference between the Finn cycling index measured in Barabási-Albert type scalefree networks and the Erdő-Rényi type random networks for a given density and openness

The difference between the asymmetric and symmetric structures in the Finn cycling index is shown in Fig. 3, where the previous exercise was done for different combinations of openness (as in Eq. 5) and network density. Network size in all calculations was $N=100$.

**Fig. 4:**
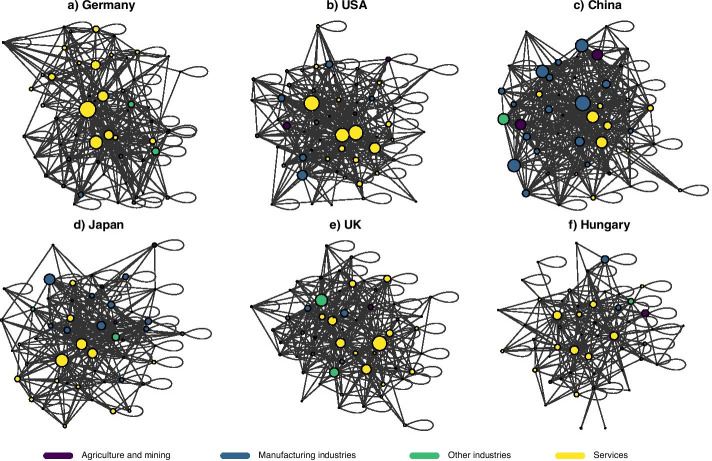
Six different domestic production networks. The size of the nodes reflect the importance of sectors, measured by their out-degree centrality. The coloring refer to broad sector categories (dark magenta: agriculture and mining, blue: manufacturing industries, green: other industries, yellow: services). The figure only shows those edges where the input share between two sectors is larger than 1.5%. **a** Germany, **b** USA, **c** China, **d** Japan, **e** UK, **f** Hungary

As the edge weights in these networks are all 1, it is only the difference in network structures (asymmetric vs. symmetric) that shapes the value of the Finn cycling indices for given openness and density. It is clear that an asymmetric network structure always gives a higher cycling index then a symmetric one. While this difference is very pronounced for low openness and moderate densities, it vanishes as we move towards the empty or the complete network as well as towards full openness. The latter conclusions are plausible: there is no difference between empty networks with respect to their structure (symmetry) and the same is true for complete networks. Also, there is relatively minor role for the structure in very sparse and very dense networks. The true difference between symmetric and asymmetric structures (degree distributions) can unfold under moderate densities. On the other hand, as the domestic transaction links lose their importance when moving towards extreme openness, the relevance of domestic asymmetry also vanishes as these links play a marginal role in the whole economy.

### Data

We have introduced the methodological framework for this study so far. This requires information on domestic input–output relationships as reflected by the transaction matrix ${\mathbf {W}}$ and data on imports, value added (possibly taxes and transport margins), final use and exports. In this study, we reach out to the World Input–Output Database (WIOD 2016) (Dietzenbacher et al. 2013; Timmer et al. 2015), which supplies this information on a relatively wide set of countries (43) and sectors (56) and for a considerable long time period on an annual basis (15 years between 2000 and 2014). The list of countries and sectors included in the analysis can be found in the “Appendix 2”. We use the US dollar transaction volumes in these data tables as the primary source to feed the model framework established in the previous subsection. Also, these tables contain information on total output, import and export as well as value added which is used to calculate the *FCI* values as given above.

While the original WIOD dataset explicitly takes into account international transaction on a country-sector basis, as our focus is on the structure of domestic production networks, we simply add these transactions in vector ${\mathbf {m}}$ as the overall external exposure of the domestic sectors.

In addition to the data background of the *FCI* calculations, which is based on the WIOD 2016, further empirical analysis builds on some macroeconomic time series of the countries involved in our sample, including GDP per capita, population, capital stock and labor intensity. These are retrieved from the Penn World Tables (version 9.1) released by the University of Groningen (Feenstra et al. 2015). The table contains several macroeconomic data in a wide range of countries and time, but we use only a few of these, which are reported with their descriptive statistics in Table 2.

**Table 2:** The descriptives statistics of variables

Variable	Data source	Min	Max	Mean	Stand.dev.
GDPPC	PWT 9.1	0.0025	0.0675	0.0320	0.0180
(in million 2011 US$, PPPs)					
Population	PWT 9.1	0.3967	1390.11	100.72	264.66
(in million)					
AvgWorkedHours	PWT 9.1	1362.6976	2509.2881	1816.8754	223.6219
(average annual hours worked by persons engaged)					
Capital	PWT 9.1	0.0202	77.7446	5.5357	10.0540
(in thousands billion 2011 US$, PPPs)					
Finn cycling index (*FCI*)	WIOD 2016	0.0250	0.1909	0.0865	0.0289
(percentage, own calculation)					
Openness ($$\tilde{m}$$)	WIOD 2016	0.0556	0.6006	0.2300	0.1108
(percentage, own calculation)					
Linkskew	WIOD 2016	5.5516	15.2338	8.5071	1.5560
(own calculation)					

In order to account for the effect of being part of the European Union and the Eurozone in further empirical investigations, we construct specific dummy variables. The value of the EU dummy is 1 if the country is a member of the European Union for more than six months in a given year. Similarly, the EURO dummy is 1, if the country is a member of the Eurozone for more than six months in a given year. In other cases, both dummies are 0.

## Results

In this section, we present the results of the analysis that we carried out on the basis of the previously introduced methodological framework. According to this framework, we pay attention to the structure of domestic production networks from three interrelated angles: openness (i.e. foreign exposure), systemic operation (the *FCI* measure) and asymmetry. First, we present some descriptive results with respect to the asymmetry and openness of the economies. Then, we turn to the analysis of systemic operation. In this respect, we provide a description of the raw *FCI* scores, and then estimate the effects of asymmetry, openness and other macroeconomic variables in shaping these scores. Here, we argue that using adequate econometric techniques, one can clear the *FCI* from those economic characteristics which naturally influence these characteristics.

### Asymmetry and openness

In this subsection, we provide an analysis of domestic production networks with respect to their asymmetry and openness. In Fig. 4, we highlight six countries and their production networks. The nodes are the 56 sectors that are included in the WIOD database, while the edges represent the relative input shares (the entries from matrix ${\mathbf {A}}$). The size of the nodes reflect the importance of sectors, as measured by their out-degree centrality ($\sum _{j}a_{ij}$), and coloring shows the broad category of the sectors. For a better visualization, only those edges are shown in the figure which are larger than 1.5% in terms of the input-share of a given sector ($a_{ij}>0.015$). For similar reasons, vertices that thus have no connections were not displayed. Although edges are directed, we do not show arrows and present them overlapping for easier representation. The links in the graph are visualized as unweighted.

These graphs demonstrate that the sectors play different, asymmetric roles in these production networks. Although some sectors have a central position in the production process, most of them have significantly less importance. This is consistent with what has been found in previous studies in the literature (Acemoglu et al. 2012; Contreras and Fagiolo 2014). Edge weights follow a similar pattern: sectors typically has a few outstanding suppliers, while their connection to others are relatively weaker. Strong self-loops represent that sectors use their own output to a relatively large extent—which is explained by the level of aggregation given in the WIOD data structure.

Another important finding is that the countries’ production networks are very heterogeneous in terms of the relatively important/central sectors. For example, in the case of Germany, the most important sectors contributing to production are the service industries, such as the administrative and support activities, warehousing and support activities for transportation, and real estate activities. In China, though, the manufacturing industries, such as the manufacture of coke and refined petroleum products, chemicals and chemical products as well as basic metals have a larger out-degree centrality, similar to two agriculture and mining sectors (crop and animal production, mining and quarrying).

**Fig. 5:**
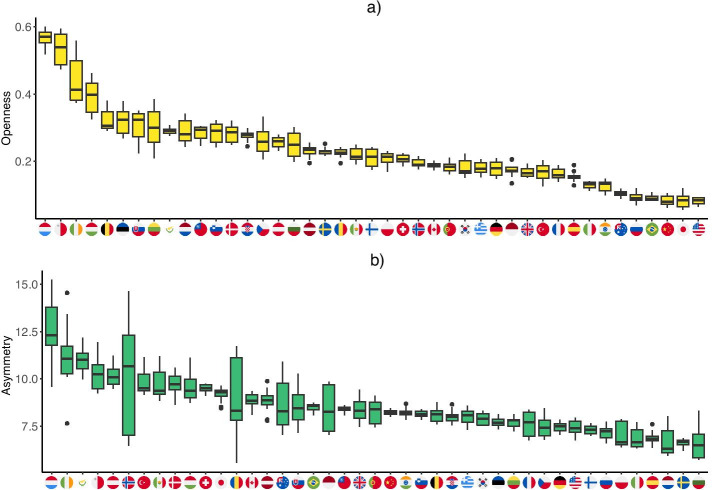
The asymmetry and openness of countries’ domestic production networks. The box plots present the values between 2000 and 2014 by countries, ordering by the mean values. **a** Openness, **b** Asymmetry

A further finding is that the average weight of links of these networks is very different. The Chinese sectors are more closely related than those in the other countries, especially in Hungary, where there are significantly fewer dominant supplier relationships. For the former, the average weight of links is 0.0089, while for the latter, it is only 0.0050. The reason is that the Hungarian economy is a very open economy and the inputs come from foreign sources to a relatively large extent, weighting down domestic input transactions in a high ratio. The upper part of Fig. 5 shows that Hungary is the fourth most open country in our sample and approx. 40% of inputs originate from outside the country. The most open countries are the small economies, such as Luxembourg, Malta, and Ireland. By comparison, the Chinese, the Japanese, and the US sectors source less than 20% of their inputs from abroad, resulting in a lower foreign exposure.

These illustrative results about the six highlighted countries point to the importance of heterogeneity and asymmetry in network structure, as well as the role of openness. In what follows, we build strongly on these aspects of network structure. Figure 5 shows how openness and structural asymmetry varies in the countries in our sample. The box-plots in this figures represent the distribution of annual openness and asymmetry (skewness) values at the country level.

Figure 5 provides a few important insights into the country-level heterogeneity in openness and asymmetry. First, there is strong heterogeneity in both respects. Although openness and its connection to size is well documented in the literature, the picture in Fig. 5 shows that cross-country variation is significantly larger than within-country variation over time. The level of asymmetry, as measured by the skewness of the input-share distributions, proves to be significant and we observe strong heterogeneity across countries. The values of observed skewness show a significant positive or right-skew in the case of all countries. This reflects an asymmetry biased towards relatively low-weight connections, from which a few important high-weight connections stand out. Here, variance over time tends to be relatively large for some countries (Norway, Romania, Indonesia, Australia), while heterogeneity across countries is large, the difference between average values in the lowest and highest scoring countries (Bulgaria and Luxembourg respectively) being almost two-fold. Although at the top of the two lists we find the same countries (Luxembourg, Ireland, Malta, Cyprus and Hungary), the correspondence between the two rankings gets loose beyond these few top countries. This shows that extreme openness goes together with extreme structural asymmetry, but for more moderate values we do not find correlation. This is reinforced by the observation of correlation coefficients between the two variables. The standard Pearson correlation coefficient is moderate (0.46), while the Spearman rank correlation is low (0.29). This reveals that the large values in asymmetry and openness for the few countries mentioned above outweight the uncorrelated majority: correlation is only present at the first few ranks of the variables. Finally, both indicators seem to be quite stable over time.

### Systemic operation of the domestic production networks

In this subsection, we go further in exploring the structure of the domestic production networks and employ the cycling index as defined in Eq. 4 to describe the extent to which countries are able to systemically operate in terms of their production network structure. We use the WIOD 2016 to feed the model set out previously. First, we give a brief description of the resulting cycling index scores and then analyze how different sectors contribute to these scores by country.

As it was argued earlier, the systemic operation of an economy can be described by the Finn cycling index, which measures the strength of cycling (feedback loops) through the direct and indirect linkages among sectors. Countries with a higher score in this respect build more heavily on intermediate inputs from other sectors not only directly, but also indirectly through feedback loops and mutual connections. In the first step, based on Eq. 3, we can determine the contribution of a sector to the systemic operation. In the second step, we can calculate the country-level cycling index through a weighted average as in Eq. 4.

**Fig. 6:**
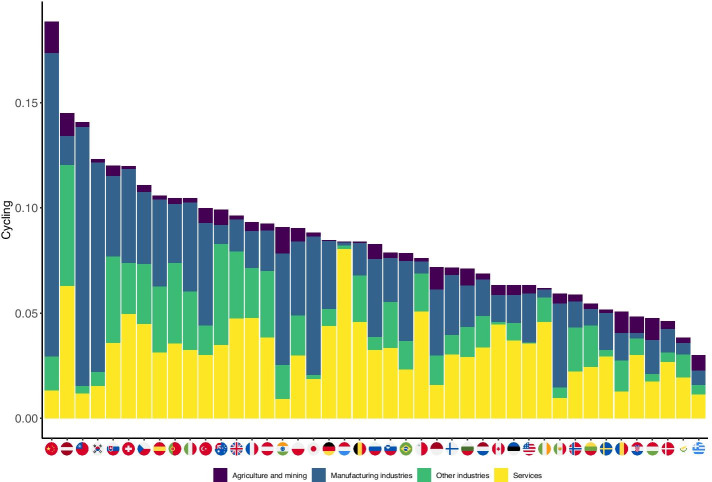
The countries’ Finn cycling index in 2014. The colors represent the contribution of different types of sectors (dark magenta: agriculture and mining, blue: manufacturing industries, green: other industries, yellow: services)

Figure 6 presents the country level cycling indexes by sectoral decomposition based on data available for 2014 (the latest year in the sample). The decomposition reflects broad sector categories as defined in Table 5 presented in the “Appendix 2”. The results show that the overall, country-level cycling index (i.e. the strength of systemic operation) is highest in China (0.1884), and it is six times larger than the lowest value found for Greece (0.0300). Latvia (0.1449), Taiwan (0.1407), and the Republic of Korea (0.1200) follow China according to this ranking, while Hungary (0.0475), Denmark (0.0468) and Cyprus (0.0384) are found at the end of the ranking in addition to Greece. The sectoral decomposition again shows marked heterogeneity across countries. The different broad sectors have different relative weights in contributing to the country-level cycling index. For instance, Chinese manufacturing industries determine the country’s cycling index by 76%. These sectors also play an important role in Taiwan (87%), the Republic of Korea (81%) and Japan (77%). Other manufacturing industries, such as construction, determine systemic operation to a large extent in Australia (49%), Latvia (40%) and Portugal (37%). Service sectors contribute significantly to country-level cycling, among others, in Luxembourg (96%), the Netherlands (49%) and Malta (38%). Although agriculture and mining are not among the largest contributors in any of the countries, it has a relatively dominant role in Hungary (22%), Romania (20%) and Croatia (17%).

The impression from Fig. 6 further shades our previous findings about the structural heterogeneity of domestic production networks. The cycling index varies in a broad range pointing to very different capacities within national economies for systemic operation. Also, countries are quite diverse with respect to the sectors which contribute to the cycling index, i.e. the sectors which are better embedded into, contribute more to the productive activities within the whole domestic economy. These findings also coincide with the observations from Fig. 4. There, we have seen that Hungary has a less dense domestic production network than e.g. China. With respect to the structural properties, we then see that the Hungarian production network is relatively open and asymmetric, compared to the five other countries displayed in Fig. 4. These observations point to the role of these structural properties as well as other (macro-)economic variables to play a role in the value of the country-level cycling index. In the next subsection we elaborate on this issue.

### The role of structural and macroeconomic properties in shaping the cycling index

In order to further explore and understand the determinants of the cycling index, we carried out an econometric exercise where the country-level Finn cycling indices are introduced as dependent variable while structural and other macroeconomic variables are used as independent variables. We utilize the panel setup of our data, i.e. that we have information on countries annually over a relatively long period of time. We use the *FCI* as introduced in Eq. 1 on the left hand side, while openness as in Eq. 5 and skewness of the input shares in matrix ${\mathbf {A}}$ for all countries and years are the focus variables on the right hand side. Other macroeconomic control variables include GDP per capita, population, average hours worked and capital stock. The source of these control variables is the Penn World Database and the structural measures (cycling, openness and asymmetry) are based on the WIOD 2016 version. Finally, two dummy variables are used to control for the membership of countries in the Euro Area and the European Union. Descriptive statistics of the data series employed are given in Table 2. Building on the panel nature of the data, we set up the following general empirical model between the cycling index and the independent variables:

6 $$\begin{aligned} FCI_{c,t} = \mathbf {\alpha }^{T} {\mathbf {z}}_{c,t} + \beta _{1} {OP}_{c,t} + \beta _{2} {SK}_{c,t} + \mu _{c} + \tau _{t} + \varepsilon _{c,t} \end{aligned}$$

In this equation the vector ${\mathbf {z}}_{c,t}$ denotes the set of (macroeconomic) control variables for country *c* in period *t* and $\mathbf {\alpha }$ is the corresponding vector of coefficients. $OP_{c,t}$ then labels openness while $SK_{c,t}$ is the skewness of domestic input shares for country *c* in period *t*. $\mu _{c}$ is a country-specific fixed effect, $\tau _{t}$ is a time-period fixed effect while $\varepsilon _{c,t}$ is an observation-specific error term. The two fixed effects can be estimated in a panel setup and they allow to control for unobserved heterogeneity in the specific countries and time periods respectively.

We apply the following strategy to estimate the model in Eq. 6. First, we include only the control variables in ${\mathbf {z}}_{c,t}$ on the right hand side as a baseline model. These include the macroeconomic variables and the dummies for EU and EA membership. The intuition behind the variables included is that the level of development (measured by GDP per capita), size (measured by population) and the factors of production used (capital and hours) are all characteristics of a country’s economy which may influence the level of systemic operation. More developed countries can be involved in the international division of labor in more sophisticated ways which affects their internal structure of production networks as well. Larger countries on the other hand tend to be more self-sufficient, thus one expects a higher level of systemic operation in their case. On the other hand, common market and currency as measured by the EU and EA dummies are expected to affect the way how global production networks are organized, thus domestic production networks as well—specifically we expect that motivating more intensive trade between member countries, these areas lead to a lower level of systemic operation within borders.

Model 0 and Model 1, as shown in Table 3, provide estimates of this baseline setup. In Model 0 we use a pooled OLS estimation, which do not take into account the panel structure of the data and as a result, it does not estimate fixed effects $\mu _{c}$ and $\tau _{t}$. This can serve as a reference point for further, more accurate estimations with fixed effects. Model 1 still includes only the control variables on the right hand side, but it is a fixed effects panel estimation. In the next step, we expand the model by the structural properties of the domestic production networks separately (Model 2 and Model 3). Then, we include openness and asymmetry among the independent variables together (Model 4).

**Table 3:** The results of panel regression models

	Model 0	Model 1	Model 2	Model 3	Model 4
	Pooled OLS	FE Panel	FE Panel	FE Panel	FE Panel
Intercept	0.05133***				
	(0.01044)				
GDPPC	0.16931**	− 19.62148	− 10.38289	− 22.75483	− 13.72812
	(0.07148)	(18.16963)	(18.42629)	(16.79573)	(17.02245)
Population	0.00003***	− 0.00017***	− 0.00016***	− 0.00016***	− 0.00014***
	(0.00001)	(0.00002)	(0.00002)	(0.00002)	(0.00002)
AvgWorkedHours	0.00001***	0.00004**	0.00003**	0.00004*	0.00002*
	(0.00001)	(0.00002)	(0.00001)	(0.00002)	(0.00002)
Capital	− 0.00005	0.00099***	0.00083***	0.00100***	0.00083***
	(0.00015)	(0.00016)	(0.00020)	(0.00015)	(0.00019)
EU	− 0.00631**	− 0.00766***	− 0.00483	− 0.00694***	− 0.00356
	(0.00293)	(0.00278)	(0.00325)	(0.00255)	(0.00252)
EURO	0.01019***	0.00195	0.00150	0.00161	0.00099
	(0.00304)	(0.00238)	(0.00195)	(0.00241)	(0.00187)
Openness			− 0.13531***		− 0.14901***
			(0.04687)		(0.04672)
LinkSkew				0.00148**	0.00202***
				(0.00066)	(0.00078)
Country.FE	No	Yes	Yes	Yes	Yes
Year.FE	No	Yes	Yes	Yes	Yes
Observations	645	645	645	645	645
Adj.R$^2$	0.1385	0.1839	0.2928	0.2071	0.3364

White-standard errors is parantheses, $$***<0.001, **<0.05, *<0.1.$$

Table 3 presents the results of the five different models. The null-model with pooled regression (Model 0) shows significant effects for almost all included control variables, however, these estimations are possibly biased due to the potentially omitted variables. This problem can be minimized by the fixed effect panel estimation in Model 1, where the estimated country and time fixed effects control for unobserved heterogeneity. Comparing the two models, we can see that inclusion of the fixed effects in the model has considerably changed the results. The coefficient of the population becomes negative and larger than in Model 1, while the effect of GDP per capita and membership in the Eurozone becomes insignificant. Intuition would suggest that a larger country is capable of a more systemic operation because the size of the market and availability of resources allows it to be more self-sufficient, with a stronger interconnection between domestic sectors. The results show that this is true for capital, while it isn’t true for population. Better availability of capital allows for the production of more complex products which requires a more sophisticated division of labor, thus production network. On the other hand, population reflects the mere size of the country without any reference to productivity. Larger population comes with a larger population of economic units as well, thus one expects the individual (company-) level transaction network to be more sparse. Although we observe networks at a more aggregated level (56 sectors), still these aggregated network connections are representations of the micro-level transaction networks. In addition, we find a positive significant effect for average annual hours worked. This measure reflects the average annual labor input, normalized by the size of workforce (which strongly correlates with population, hence the normalization). In other terms, we reflect the intensive margin of labor use: how intensively the given country uses its available workforce. Finally, the common market as proxied by the EU dummy has a negative significant effect on cycling as expected: trade agreements reduces the barriers to trade, which shifts production network connections towards other members, and decrease the role of domestic transactions.

In the rest of the models we also include either openness or asymmetry or both among the independent variables. Thus, these models are useful in assessing the role of these two structural factors in shaping the cycling index, i.e. the capacity of economies for systemic operation. As expected after the short analytical exercise in the methodology section, openness indeed seems to decrease cycling in domestic production networks, which is an intuitive result.

On the other hand, asymmetry has a positive significant effect on cycling: the more skewed domestic input shares are, the more capable countries are to operate in a systemic way. This result is puzzling, as previous studies suggest that the higher asymmetry in production networks leads to higher volatility in aggregate output (Acemoglu et al. 2012), which doesn’t seem to be a sign of systemic operation. The solution to this puzzle may come from observing the microstructure of these networks. In the case of a balanced, symmetric network/economy with no outstanding connectivity at a few nodes/sectors, the share of closed triangles, i.e. clustering, is lower. In other words, the direct neighbours of sectors are only connected to each other to the extent as the whole network is connected. In contrast, the share of closed triangles (clustering) is shown to be higher in a scale-free, i.e. asymmetric network. As the cycling index detects the strength of feedback loops (as shown in the methodology section), this overrepresentation of closed triangles or feedback loops will result in a higher cycling index.

The positive significant effect of asymmetry and negative significant effect of openness remains if the two variables are included together in the estimation (Model 4), and the estimated coefficients have similar magnitudes. The other control variables mostly keep their role compared to Model 1. Population remains negative and strongly significant, average hours worked looses part of its significance, while capital remains strongly significant. The estimated coefficients also seem to be robust across these estimations. The EU dummy, though loses its significance when the variable openness is included in the model (Model 2 and Model 4). The reason for this is that being a member of the European Union naturally leads to more openness, so the effect of EU membership estimated in Model 1 and Model 3 is captured by the Openness variable in Model 2 and Model 4. Finally, due to the moderate correlation between asymmetry and openness, we also checked the multicollinearity between these variables. The VIF parameter of asymmetry in Model 4 is 1.43, while in the case of openness, it is 2.10. This suggests that there is no significant multicollinearity in the model, hence the estimations of coefficients are robust.

**Fig. 7:**
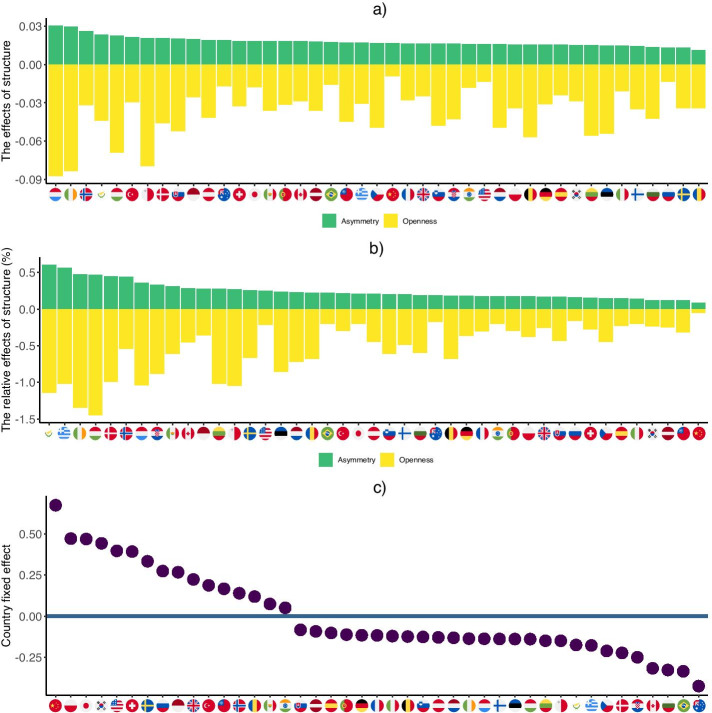
The effects of structural properties on Finn cycling index. **a** the absolute effects of asymmetry and openness on Finn cycling index in 2014, **b** the relative effects of asymmetry and openness on Finn cycling index in 2014, **c** the country fixed effect relative to the mean value

Figure 7 shows the contribution of openness and asymmetry to the Finn cycling index in absolute and relative terms, based on 2014 data. These charts are obtained by using the estimated Eq. 6 according to Model 4. Observed openness ($OP_{c,t}$) and asymmetry ($SK_{c,t}$) are multiplied by their respective estimated coefficients ($\beta _{1}$ and $\beta _{2}$). These values are reported in panel (a) of Fig. 7. The relative importance in the overall cycling index is shown in panel (b), where the previously described absolute contributions are normalized by the value of the cycling index. The resulting rankings show interesting results. An important conclusion is, that the contribution of asymmetry and openness vary across countries. An interesting example is China, which has the largest cycling index (see Fig. 6), but most of its value is attributed to its capital stock, hours and population while the structure of domestic production networks play a very minor role. Also, we may recall from Fig. 6 that the strength of systemic operation in the Hungarian economy is relatively low. Although the Hungarian production network is very asymmetric, and this property increases the strength of systemic operation, openness has a much larger negative effect on its value. Similarly, in the case of Cyprus, Greece, Ireland and Denmark, the Finn cycling index is low because of the strong openness.

### Towards a clarified measure of systemic operation

In the previous subsection, we have presented the results from a few model estimations where the Finn cycling index was explained by several (macro-)economic indicators as well as the structural properties of the domestic production networks, namely openness and asymmetry. Most of these variables characterizing a country’s economy proved to affect significantly the cycling index, i.e. the capability for systemic operation. On the other hand, this result also means that the raw scores of the cycling index as presented in Fig. 6 contain the effect of several factors which shape the capability of systemic operation of countries: their mere size, availability of production resources, openness and asymmetry. Having this in mind, it is not surprising that China turns out to be at the top of the ranking with respect to the cycling index: being the largest country, it has an opportunity to efficiently develop self-sufficient production systems. However, one may ask how systemic the Chinese economy is *given* its size, level of development, openness, etc.

The econometric technique used above, namely fixed effect panel estimation provides a straightforward answer to this question. These estimations provide an estimate of the country fixed effects, i.e. $\mu _{c}$ in Eq. 6. By the construction of this method, these fixed effects capture all heterogeneity in the dependent variable (the *FCI*), which is not accounted for by the other covariates on the right hand side and constant over time. As a result, these fixed effects are good candidates to measure that part of cycling, what is not explained by the independent variables included in the model.

In other terms, using fixed effect estimates from Model 4 can show the extent of systemic operation which is specific to a given country, and can not be attributed to openness, asymmetry, population, hours and capital. Panel (c) in Fig. 7 presents the country fixed effects estimated in Model 4. In order for a clear interpretation, we subtracted the average of these fixed effects from the value of all countries, thus the figure shows the deviation of the fixed effects from their mean (which can be regarded as an overall constant term in the regression).

Looking at the picture of these fixed effects, the most important observation is that the ranking of countries can significantly change compared to the raw cycling indices presented in Fig. 6. Although China keeps its top position, its relative advantage over the second rank is smaller. Also, Poland and Japan come up from the mid-range of the cycling index to the top, while the US arrives 5th after a position in the last third with respect to the cycling index. This means that these economies show a relatively high level of systemic operation even if we rule out those natural conditions which proved to be important in shaping the cycling index. In other terms, this ranking is able to draw attention to those countries which show some additional extent of cycling or systemic operation inherent in their domestic production networks, which is not attributable to their size, factor endowment, openness or structural asymmetry.

## Discussion and conclusion

In this paper, we examined the systemic operation of domestic production networks and showed how structural properties, such as openness and asymmetry, affect this systemic operation. The structure of the domestic production network is different across countries in terms of sectors’ centrality and the intensity of the linkages between domestic sectors. The fixed effect panel regressions revealed that the other country-specific properties also play an important role in shaping systemic operation.

The level of systemic operation measured by the Finn cycling index can be a useful signal of how resilient an economy is to emerging global shocks such as a pandemic, natural disasters or wars. For example, lockdowns triggered by the COVID-19 pandemic may hit those countries more harshly which are characterized by lower levels of systemic operation because it is challenging for local companies to replace foreign inputs with domestic substitutes. The over-dependence on trade relations and the lack of strong interconnectedness among domestic economic actors, as reflected by the Finn cycling index, makes an economy vulnerable to this type of shock. However, our dataset is not suitable to directly analyze the recent pandemic due to its time coverage. Still, further research should clearly focus on this issue in more detail.

The ranking of systemic operation shows the Chinese economy relies the most on domestic inputs. However, the previous studies in the literature suggest that the Chinese value-added ratio of gross export is similar to the US (Johnson and Noguera 2012; Timmer et al. 2015). The reason for this seeming contradiction that the Chinese economy is basically involved in the lower stages of the production phase, processing basic raw materials and carrying out simpler, less technologically demanding production (Cingolani et al. 2017). In contrast, the U.S. economy relies less on domestic inputs, but performs higher stages of production phases, resulting in relatively higher value-added production. The manufacture of smartphones gives a good example of this production process. The devices are mainly manufactured in China, but innovation and software development take place in the US.

On the other hand, the other interesting result is the positive effect of asymmetry on the systematic operation. Acemoglu et al. (2012) showed that the higher asymmetry of structure can lead to higher volatility in aggregate output. It means that the shock propagation between sectors is powerful in an asymmetric economy. This process may be based on the fact that the sectors rely more on each other’s input products in the case of an asymmetric structure, in other words, they are more interdependent. This result confirms that renationalizing can reduce risks from abroad, but at the same time, raises the risks of the domestic economy (Bonadio et al. 2020).

A methodological deficiency of this study is the question of the gross and net input–output table, shortly discussed in the study. The assumption that the intrasectoral transactions are basically intersectoral, needs deeper analysis. Additionally, the level of aggregation might alter the results about the asymmetry of sectors. Similarly, the separation of the effects of openness, asymmetry, and other macroeconomic variables can be performed in different ways and the suggested method of this paper might not be the best one. All these deficiencies at the same time are further research areas. However, apart from these starting difficulties of the usage of the toolbox of ecological network analysis, results shed light on new aspects of the nature and effect of the international supply chain.

## Data Availability

All data can be downloaded free of charge online. WIOD: http://www.wiod.org/database/wiots16. Penn World Tables: https://www.rug.nl/ggdc/productivity/pwt/pwt-releases/pwt9.1.

## Appendix

### Appendix 1: derivation of general cycling index values for the two extreme cases

In the first extreme case, the transaction matrix ${\mathbf {W}}$ is the identity matrix with zeros on its main diagonal and zeros elsewhere. In these derivations, without restricting the generality of the results, we assume $v_{i}=0$ $\forall i$, and $m_{i} = m$ $\forall i$. As a result, we get $x_{i} = 1 + m$ for all sectors *i*. According to the definition in 1, the diagonal elements of matrix ${\mathbf {A}}$ are identically $1/(1+m)$, while we have zeros elsewhere. Finally, the Leontief-matrix ${\mathbf {L}}$ in this case contains $1-1/(1+m)=m/(1+m)$ on its main diagonal and zeros elsewhere.

In the second extreme case, the transaction matrix contains ones at all of its entries. Assuming again $v_{i}=0$ $\forall i$, and $m_{i} = m$ $\forall i$, we have $x_{i}=N + m$ for all sectors *i*. The Leontief-coefficients in ${\mathbf {A}}$ turn out to be $1/(N+m)$ at all entries, while the Leontief-matrix ${\mathbf {L}}$ has $(N+m-1)/(N+m)$ on its main diagonal and $-1/(N+m)$ elsewhere.

These calaculations show that in both cases the Leontief-matrix ${\mathbf {L}} = {\mathbf {I}} - {\mathbf {A}}$ has the general form where we have identical values *a* on the main diagonal and also identical values *b* off the main diagonal. As a result, solving for the Leontief-inverse ${\mathbf {L}}^{-1}$ requires the solution of the following matrix equation:

$$\begin{aligned} \left[ { \begin{array}{cccc} a &{} b &{} \cdots &{} b \\ b &{} a &{} \cdots &{} b \\ b &{} b &{} \cdots &{} b \\ \vdots &{} \vdots &{} \ddots &{} \vdots \\ b &{} b &{} \cdots &{} a \\ \end{array} } \right] \left[ { \begin{array}{cccc} k &{} h &{} \cdots &{} h \\ h &{} k &{} \cdots &{} h \\ h &{} h &{} \cdots &{} h \\ \vdots &{} \vdots &{} \ddots &{} \vdots \\ h &{} h &{} \cdots &{} k \\ \end{array} } \right] = \left[ { \begin{array}{cccc} 1 &{} 0 &{} \cdots &{} 0 \\ 0 &{} 1 &{} \cdots &{} 0 \\ 0 &{} 0 &{} \cdots &{} 0 \\ \vdots &{} \vdots &{} \ddots &{} \vdots \\ 0 &{} 0 &{} \cdots &{} 1 \\ \end{array} } \right] \end{aligned}$$

In the equation above, we know the values *a* and *b*, while the entries (*k* and *h*) in the inverse matrix are unknown. Due to the specific structure of the original matrix, the inverse matrix has the same structure with identical values on the diagonal and off-diagonal separately.

This matrix equation defines $N^2$ linear equations in *k* and *h*, but these collapse into two different (independent) equations determining the diagonal (*k*) and off-diagonal (*h*) elements of the inverse matrix:

$$\begin{aligned} ak + (N-1)bh= & {} 1\\ bk + ah + (N-2)bh= & {} 0 \end{aligned}$$

From the first equation we have

$$\begin{aligned} h = \frac{1}{(N-1)b} - \frac{a}{(N-1)b}k \end{aligned}$$

Substituting this into the second equations yields:

$$\begin{aligned} k = \frac{1}{a-b} - \frac{b}{(a-b)[a+b(N-1)]} \end{aligned}$$

Going back to the two extreme cases, in the first case economy we have $a=m/(1+m)$ and $b=0$. Using the results for the inverse matrix above, the Leontief-inverse ${\mathbf {L}}^{-1}$ in this case have $k=1/a=(1+m)/m$ on its main diagonal and zeros elsewhere. Using Eq. 3 the sector-level cycling index becomes

$$\begin{aligned} {\hat{l}}_{i} = 1 - m/(1+m) = 1/(1+m) = 1 - \tilde{m} \end{aligned}$$

for all sectors *i*, and in the last equation we used the normalized openness as in Eq. 5.

In the second extreme case, with the balanced economy, we have $a=(N+m-1)/(N+m)$ and $b=-1/(N+m)$. Substituting these values into the former solution for the Leontief-inverse we have $k=(m+1)/m$. It follows that the sector-level cycling indices become

$$\begin{aligned} {\hat{l}}_{i} = \frac{1}{1+m} = \frac{1 - \tilde{m}}{1+\tilde{m}(N-1)} \end{aligned}$$

### Appendix 2: sample countries and sectors

Tables 4 and 5.

**Table 4:** The list of countries in WIOD

Australia	Estonia	Latvia	Russia
Austria	Finland	Lithuania	Slovakia
Belgium	France	Luxembourg	Slovenia
Bulgaria	Germany	Malta	Spain
Brazil	Greece	Mexico	Sweden
Canada	Hungary	Netherlands	Switzerland
China	India	Norway	Turkey
Croatia	Indonesia	Poland	Taiwan
Cyprus	Ireland	Portugal	United Kingdom
Czech Republic	Italy	Republic of Korea	United States of America
Denmark	Japan	Romania

**Table 5:** The list of sectors in WIOD

Nr.	Sectors	Groups
1	Crop and animal production, hunting and related service activities	Agriculture and mining
2	Forestry and logging	Agriculture and mining
3	Fishing and aquaculture	Agriculture and mining
4	Mining and quarrying	Agriculture and mining
5	Manufacture of food products, beverages and tobacco products	Manufacturing industries
6	Manufacture of textiles, wearing apparel and leather products	Manufacturing industries
7	Manufacture of wood and of products of wood and cork, except furniture; etc.	Manufacturing industries
8	Manufacture of paper and paper products	Manufacturing industries
9	Printing and reproduction of recorded media	Manufacturing industries
10	Manufacture of coke and refined petroleum products	Manufacturing industries
11	Manufacture of chemicals and chemical products	Manufacturing industries
12	Manufacture of basic pharmaceutical products and pharmaceutical preparations	Manufacturing industries
13	Manufacture of rubber and plastic products	Manufacturing industries
14	Manufacture of other non-metallic mineral products	Manufacturing industries
15	Manufacture of basic metals	Manufacturing industries
16	Manufacture of fabricated metal products, except machinery and equipment	Manufacturing industries
17	Manufacture of computer, electronic and optical products	Manufacturing industries
18	Manufacture of electrical equipment	Manufacturing industries
19	Manufacture of machinery and equipment n.e.c.	Manufacturing industries
20	Manufacture of motor vehicles, trailers and semi-trailers	Manufacturing industries
21	Manufacture of other transport equipment	Manufacturing industries
22	Manufacture of furniture; other manufacturing	Manufacturing industries
23	Repair and installation of machinery and equipment	Manufacturing industries
24	Electricity, gas, steam and air conditioning supply	Other industries
25	Water collection, treatment and supply	Other industries
26	Sewerage; waste collection, treatment and disposal activities; materials recovery	Other industries
27	Construction	Other industries
28	Wholesale and retail trade and repair of motor vehicles and motorcycles	Services
29	Wholesale trade, except of motor vehicles and motorcycles	Services
30	Retail trade, except of motor vehicles and motorcycles	Services
31	Land transport and transport via pipelines	Services
32	Water transport	Services
33	Air transport	Services
34	Warehousing and support activities for transportation	Services
35	Postal and courier activities	Services
36	Accommodation and food service activities	Services
37	Publishing activities	Services
38	Motion picture, video and TV programme production, sound recording and music publishing activities; etc.	Services
39	Telecommunications	Services
40	Computer programming, consultancy and related activities; information service activities	Services
41	Financial service activities, except insurance and pension funding	Services
42	Insurance, reinsurance and pension funding, except compulsory social security	Services
43	Activities auxiliary to financial services and insurance activities	Services
44	Real estate activities	Services
45	Legal and accounting activities; activities of head offices; management consultancy activities	Services
46	Architectural and engineering activities; technical testing and analysis	Services
47	Scientific research and development	Services
48	Advertising and market research	Services
49	Other professional, scientific and technical activities; veterinary activities	Services
50	Rental and leasing activities, Employment activities, Travel services, security and services to buildings	Services
51	Public administration and defence; compulsory social security	Services
52	Education	Services
53	Human health and social work activities	Services
54	Creative, Arts, Sports, Recreation and entertainment activities and all other personal service activities	Services
55	Activities of households as employers; undifferentiated goods- and services-producing activities of households for own use	Services
56	Activities of extra-territorial organisations and bodies	Services

## Abbreviations

FE: Fixed effect
GDP: Gross domestic product
OLS: Ordinary least squares
UK: United Kingdom
US/USA: United States of America
WIOD: World Input–Output Database
